# Biotic Supplements for Renal Patients: A Systematic Review and Meta-Analysis

**DOI:** 10.3390/nu10091224

**Published:** 2018-09-04

**Authors:** Anna Pisano, Graziella D’Arrigo, Giuseppe Coppolino, Davide Bolignano

**Affiliations:** 1 CNR-Institute of Clinical Physiology, Reggio Calabria 89124, Italy; pisanoanna@hotmail.it (A.P.); g.darrigostat@tin.it (G.D.); 2 Nephrology and Dialysis Unit, “Pugliese-Ciaccio” Hospital of Catanzaro, Catanzaro 88100, Italy; gcoppolino@hotmail.it

**Keywords:** chronic kidney disease, end-stage kidney disease, gut microbiota, prebiotics, probiotics, synbiotics

## Abstract

Intestinal dysbiosis is highly pervasive among chronic kidney disease (CKD) patients and may play a key role in disease progression and complications. We performed a systematic review and meta-analysis to evaluate effects of biotic supplements on a large series of outcomes in renal patients. Ovid-MEDLINE, PubMed and CENTRAL databases were searched for randomized controlled trials (RCTs) comparing any biotic (pre-, pro- or synbiotics) to standard therapy or placebo. Primary endpoints were change in renal function and cardiovascular events; secondary endpoints were change in proteinuria/albuminuria, inflammation, uremic toxins, quality of life and nutritional status. Seventeen eligible studies (701 participants) were reviewed. Biotics treatment did not modify estimated glomerular filtration rate (eGFR) (mean difference (MD) 0.34 mL/min/1.73 m^2^; 95% CI −0.19, 0.86), serum creatinine (MD −0.13 mg/dL; 95% confidence interval (CI) −0.32, 0.07), C-reactive protein (MD 0.75 mg/dL; 95% CI −1.54, 3.03) and urea (standardized MD (SMD) −0.02; 95% CI −0.25, 0.20) as compared to control. Outcome data on the other endpoints of interest were lacking, sparse or in an unsuitable format to be analyzed collectively. According to the currently available evidence, there is no conclusive rationale for recommending biotic supplements for improving outcomes in renal patients. Large-scale, well-designed and adequately powered studies focusing on hard rather than surrogate outcomes are still awaited.

## 1. Introduction

Under healthy conditions, the gut hosts more than 100 trillion microbial cells that play an active role in regulating physiology, metabolism, nutrition and even the immune function of the human body. This results from a subtle symbiotic relationship between microbiome and host, in which an imbalance may trigger or exacerbate several pathological conditions not limited to the intestinal tract, such as obesity, insulin resistance, cancer, diabetes and chronic inflammatory systemic diseases [1]. There is now accruing evidence indicating that chronic kidney disease (CKD), particularly end-stage kidney disease (ESKD), causes dysbiosis of the intestinal microbiome by increasing the presence of pathogenic flora over symbiotic bacteria. Gut pathogens enhance protein fermentation, eventually generating waste metabolites such as indoles, phenols and amines; in addition, endotoxins produced by these harmful microbes may elicit a local inflammatory response which alters the permeability of the intestinal barrier, leading to an increased absorption of toxic substances into the systemic circulation. In renal patients, such mechanisms have been called into question as key triggers towards systemic inflammation, malnutrition, uremic toxicity and even progression of CKD and associated cardiovascular (CV) disease [2].

Targeted interventions to restore symbiosis have hence been proposed and tested in this population setting for alleviating uremic symptoms and improve renal outcomes. These may include the administration of 1) prebiotics, which are non-digestible fiber compounds stimulating the growth or activity of advantageous bacteria; 2) probiotics, which are, on the contrary, live beneficial microorganisms commonly employed to improve digestive health; 3) synbiotics, which represent a synergistic combination of pre- and pro-biotics.

The mechanism by which these products exert their favorable effects may include protection of the intestinal barrier, changes in intestinal pH and suppression of pathogens by competitive exclusion and competition for available nutrients [3].

However, despite a wealth of animal studies and small uncontrolled pilot trials that evidenced a positive impact of biotic supplements towards the clinical course of CKD [4], no univocal benefits were reported by more recent randomized clinical trials focusing on a myriad of endpoints pertaining to renal function/damage, uremic toxicity or inflammation [5,6,7].

With this background in mind, we therefore felt it necessary to perform a systematic review focusing on randomized clinical evidence in order to ascertain whether chronic biotic supplementation should indeed be advocated as an additive therapeutic measure for improving outcomes of renal patients.

## 2. Methods

This review follows PRISMA guidelines [8] for reporting in systematic reviews and meta-analysis and was conducted according to a previously published protocol (PROSPERO ID: CRD42018087391).

### 2.1. Data Source and Search Strategy

Ovid-MEDLINE, PubMed and CENTRAL databases were searched for articles without time or language restriction up to 5 March 2018 using focused, highly sensitive search strategies (Supplementary Table S1). References from relevant studies and reviews were screened for additional articles. The search was designed and performed by three authors (D.B., A.P., G.C.).

### 2.2. Study Selection and Data Extraction

We aimed at including any randomized control trials (RCT) or quasi-RCT (trials in which allocation to treatment was made by alternation, use of alternate medical records, date of birth or other expected methods) testing the effects of biotic supplements (pre-, pro- or synbiotics) in patients with chronic kidney disease (CKD) or end-stage kidney disease (ESKD) on chronic renal replacement therapy by hemodialysis, peritoneal dialysis or kidney transplantation.

Studies were considered regardless of dosage of supplementation and without language and follow-up duration restrictions. Any type of comparator was contemplated, including but not limited to placebo and standard treatment. Studies comparing the same intervention employed at different doses were excluded.

The presence of CKD was defined according to the National Kidney Foundation Kidney Disease Outcomes Quality Initiative (NKF KDOQI) guidelines [9] by a reduced glomerular filtration rate (GFR) <90 mL/min/1.73 m^2^ or by the persistence of urinary abnormalities, (albuminuria, proteinuria or hematuria) in subjects with GFR ≥90 mL/min/1.73 m^2^.

The primary endpoint of interest was CKD progression, either defined as a stable increase in serum creatinine or estimated GFR (eGFR)/creatinine clearance decrease; CV mortality and morbidity (non-fatal CV events).

Secondary outcomes were change in proteinuria and albuminuria, inflammation indexes, azotemia and other uremic toxins (including but not limited to p-cresol and indoxyl sulfate), quality of life and nutritional status. 

Studies were excluded if: 1) dealing with CKD patients on acute renal replacement therapy (e.g., acute hemo- or peritoneal dialysis), 2) employing biotic supplements for other clinical indications (e.g., digestive diseases, intestinal autoimmune or infectious diseases), 3) employing an undefined combination of dietary fibers with unproven pre-biotic effects 4) not providing data on the outcomes of interest.

Studies where at least part of the population fulfilled the above criteria were included in the review.

Titles and abstracts were screened independently by two authors (A.P., G.D.), who discarded studies not pertinent to the topic. Non-randomized trials, case reports, case series, reviews, editorials, letters and studies performed on children (age <18) were excluded from qualitative analyses but screened for potential additional references. Two authors (A.P., G.D.) independently assessed the retrieved abstracts and the full text of these studies to determine eligibility according to the inclusion/exclusion criteria.

A third reviewer (D.B.) solved possible discrepancies on study judgments. Data extraction and analysis were performed by two reviewers (A.P., G.D.) and independently verified by another (G.C.).

### 2.3. Data Analysis

Cumulative meta-analyses were performed for outcomes reported, in a suitable and consistent format, by more than two studies. In order to maximize the information provided to readers, data on outcomes stated by single studies or in a descriptive way were reported narratively. The effects of treatment on continuous variables were assessed as mean difference (MD) or standardized mean difference (SMD), as appropriate. Data were pooled using the random-effects model. To ensure robustness of the model and susceptibility to outliers, pooled data were also analyzed with the fixed-effects model. Heterogeneity was assessed by the Chi-squared test on N-1 degrees of freedom, with an alpha of 0.05 considered for statistical significance and the Cochrane-I-squared (*I*^2^) statistic. *I*^2^ values of 25%, 50% and 75% were considered to correspond to low, medium and high levels of heterogeneity, respectively. Sources of heterogeneity, for identifying possible effect modifiers on the pooled analyses, were explored by sensitivity analyses according to: population characteristics, type and dose of biotic administered, study design or follow-up duration where feasible according to the number of studies matching the same characteristic.

Publication bias was investigated by the Egger’s regression test and by visual inspection of funnel plots. Statistical analyses were performed by two authors (A.P., G.D.) using Review Manager (RevMan; Version 5.3. Copenhagen: The Nordic Cochrane Centre, The Cochrane Collaboration, 2014) and Stata/IC (Version 13.1, StataCorp LP, Texas, USA).

### 2.4. Risk of Bias Assessment

Likelihood of bias in the single RCTs was evaluated by using the checklist developed by the Cochrane Renal Group which considers the presence of potential selection bias (random sequence generation and allocation concealment), performance bias (blinding of investigators and participants), detection bias (blinding of outcome assessors), attrition bias (incomplete outcome data), reporting bias (selective reporting) and possible other sources of bias (e.g., funding bias).

## 3. Results

### 3.1. Search Results

**Figure 1:**
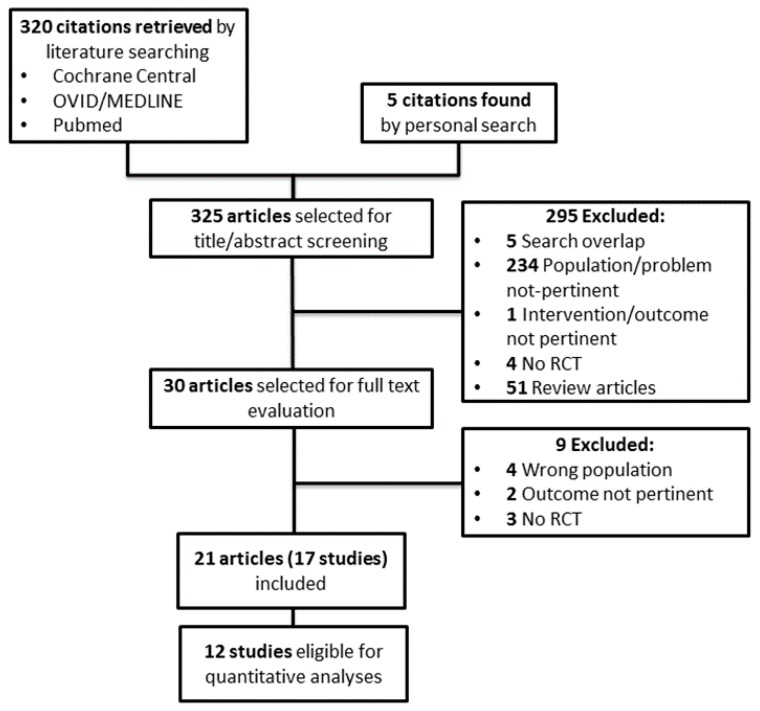
Study selection flow. RCT = randomized control trial.

Figure 1 shows the flow diagram of the study selection process. Three hundred and twenty potentially relevant references were initially found. Five additional citations were added by personal search. By screening titles and abstracts, 295 citations were excluded for various reasons (search overlap, study population or intervention or outcome not pertinent, no RCTs, review articles or other topic).

Amongst the 30 articles selected for full text examination, nine studies were excluded because: 1) no RCT (*n* = 3), 2) dealing with the wrong population (*n* = 4), 3) not providing data on the outcomes of interest (*n* = 2). 

A total of 21 articles referring to 17 studies (701 participants) were finally reviewed.

Twelve randomized trials (530 participants) providing suitable numerical data on the outcomes of interest were included in the cumulative meta-analyses. The main characteristics of these studies are summarized in Table 1.

### 3.2. Study Characteristics

All but six studies reviewed [10,11,12,14,19,20] had a parallel design. Study participants had early renal failure (NKF KDOQI stage 1–2) in two RCTs [7,24], mild-to-moderate (stage 3–4) CKD in four [5,12,13,18] and moderate-to-severe (stage 4–5) in three [11,19,20]. One study [10] recruited CKD patients with a mean serum creatinine of 4.4 ± 0.8 mg/dL with no information on GFR. One study [23] included kidney transplant recipients with stable graft function. Five studies [6,14,15,16,25] focused on hemodialysis (HD) patients and one [17] on peritoneal dialysis (PD) patients. The prevalence of diabetes spanned from 21% [17] to 100% [6,7,24] while that of hypertension ranged from 16.7% [18] to 96.7% [6]. The number of participants varied from nine [11] to 136 [7]. The mean age of patients ranged from ~40 [16] to 70 years [19]. Male gender spanned from 27% [14] to 87% [13]. Study follow-up varied from four weeks [13,19,23] to six months [12,14,17,18].

### 3.3. Risk of Bias

Risk of bias of randomized controlled trials is summarized in Table 2.

Information on the random sequence generation and allocation concealment was reported in ten [6,7,13,15,17,19,20,23,24,25] and 13 studies [5,7,10,12,13,15,16,17,19,20,23,24,25], respectively. Thirteen RCTs [5,6,7,12,13,14,16,17,19,20,23,24,25] were double blind; two studies were open label [11,18] and other two [10,15] had a single-blind design. Only two [24,25] specifically provided information on blinding of the outcome assessors. Attrition bias was low in 11 studies [5,6,10,11,13,16,17,19,20,23,24] and unclear in one [18]; five RCTs [7,12,14,15,25] reported an overall high incidence of drop-out (>21%). Reporting bias was low in all but one study [14]. Risk of funding bias was potentially high in six studies [12,14,15,16,20,24] while three other studies specifically declared any sponsor involvement [7,13,19]. No other potential source of bias was apparently present in the remaining studies.

### 3.4. Outcome Data

Data on renal function, defined as change in creatinine clearance/eGFR, was available from nine RCTs [5,6,7,11,13,17,18,20,23] while eleven studies [5,6,7,10,11,12,16,17,18,20,25] provided data on end of treatment serum creatinine. Non-fatal CV events were recorded by one study [5] while no data were available on mortality.

End of treatment proteinuria and/or albuminuria was provided by one study [20]. Among inflammation indexes, six RCTs [6,12,14,15,16,25] provided data on C-reactive protein, four studies [16,17,20,25] analyzed IL-6 levels and three studies [16,17,20] TNF-α. Changes in other chemokines, such as progranulin, IL-1β, IL-5, IL-10 and IL-17, were reported by single trials [17,20,24]. Fifteen studies [5,6,7,10,11,12,13,14,15,16,17,19,20,23,25] reported changes in azotemia and other uremic toxins. Finally, four trials [12,14,15,20] provided information on quality of life and two trials [6,16] reported patient nutritional status as a subjective global assessment (SGA) score. 

### 3.5. Effects of Biotic Supplements on Primary Outcomes

#### 3.5.1. Renal Function (GFR/Creatinine Clearance)

In a study of 39 PD patients [17], residual renal function (mL/min/1.73 m^2^) was apparently higher after six months of probiotic treatment (1.59; IQR 0.85–2.93) with respect to placebo (1.24; IQR 0.50–2.74) but no statistical comparison between groups was made. 

In another study of stage 3–5 CKD patients not on dialysis [18], GFR declined more rapidly among controls than in the active group (−11.6 ± 8.6 vs. −3.4 ± 4.6 per year; *p* < 0.001).

Conversely, another trial [13] did not report significant differences in end of treatment GFR between mild-to-moderate CKD patients receiving synbiotics or placebo.

**Figure 2:**
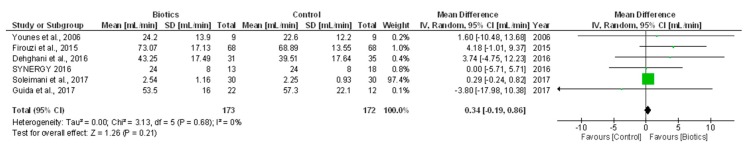
Effects of biotics supplementation vs. control treatment on eGFR/creatinine clearance; eGFR—estimated glomerular filtration rate.

This latter observation was in agreement with findings from a pooled meta-analysis of six RCTs (345 individuals) [5,6,7,11,20,23] showing no apparent effect of biotic supplementation on renal function as compared with control (MD 0.34 mL/min/1.73 m^2^; 95% CI −0.19, 0.86; Figure 2). This analysis had no heterogeneity (*Chi^2^* = 3.13, *p* = 0.68, *I^2^* = 0%) and no evidence of publication bias (Supplementary Figure S1). Study stratification by CKD stage of participants, type of biotic supplementation administered or study design (blind vs open label) did not change overall findings.

#### 3.5.2. Serum Creatinine

**Figure 3:**
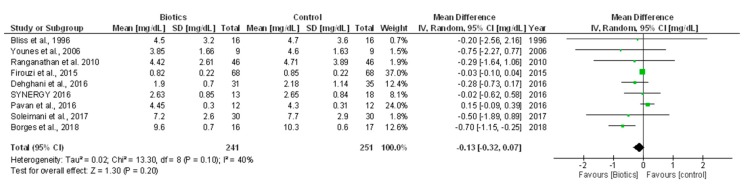
Effects of biotics supplementation vs. control treatment on serum creatinine.

In single-study data, no statistically significant differences in end-of-treatment serum creatinine were found amongst HD [16] or PD [17] patients receiving biotic supplements or placebo. These observations were in line with a cumulative meta-analysis of nine RCTs (492 individuals) [5,6,7,10,11,12,18,20,25] showing no significant change in serum creatinine after treatment with biotics versus control (MD −0.13 mg/dL; 95% CI −0.32, 0.07; Figure 3). This analysis had mild heterogeneity (*Chi²* = 13.30, *p* = 0.10; *I*² = 40%) that was totally nullified (*I²* = 0%) after excluding two studies enrolling HD patients [6,25]. Visual inspection of the funnel plot and the Egger’s regression test indicate no presence of publication bias (Supplementary Figure S1).

#### 3.5.3. Cardiovascular Morbidity

One study [5] reported one non-fatal cerebrovascular accident in a patient receiving synbiotic supplements, as compared to placebo.

### 3.6. Effects of Biotic Supplements on Secondary Outcomes

#### 3.6.1. Proteinuria/Albuminuria

In one study of hypertensive CKD patients [20], 16 weeks of synbiotic treatment significantly increased albuminuria by 38 mg/24 h (95% CI, 1 to 295 mg/24 h), although it did not affect total urinary protein excretion.

#### 3.6.2. Inflammation Indexes

**Figure 4:**
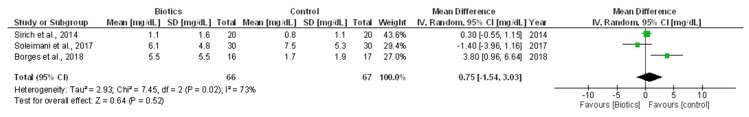
Effects of biotics supplementation vs. control treatment on C-reactive protein.

In PD patients [17], six months of treatment with probiotics significantly reduced serum levels of the pro-inflammatory cytokines TNF-α, IL-5 and IL-6 and increased those of the anti-inflammatory cytokine IL-10. Another trial [24] showed that consumption of probiotics soy milk resulted in a significant reduction (*p* = 0.01) in levels of the inflammatory adipokine progranulin as compared with placebo. Conversely, in the SYNERGY trial [20], there were no significant changes in serum concentrations of IL-1β, IL-6, IL-10 and TNF-α after synbiotic supplementation, with respect to placebo. No concrete benefits on inflammation were reported by the other two studies [14,16]. In line with these latter findings, a pooled meta-analysis of three RCTs (133 individuals) [6,15,25] reported no significant change in C-reactive protein after biotic supplementation versus placebo (MD 0.75 mg/dL; 95% CI −1.54, 3.03; Figure 4). A high level of heterogeneity was present (*Chi*^2^ = 7.45, *p* = 0.02, *I*² = 73%) that could not be further investigated given the paucity of studies included.

#### 3.6.3. Urea and Other Uremic Toxins

No differences in urea levels were reported in studies of PD [17] and pre-dialysis ESKD individuals [19] receiving a probiotic formulation as compared to placebo.

**Figure 5:**
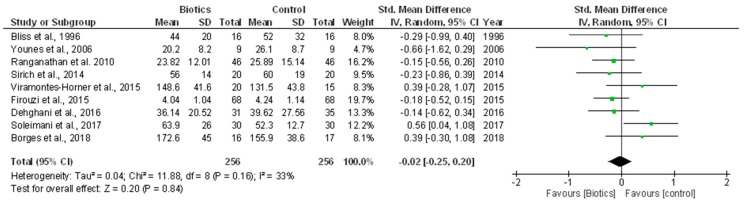
Effects of biotics supplementation vs. control treatment on urea levels.

In a pooled meta-analysis including nine RCTs (512 individuals) [5,6,7,10,11,12,15,16,25], we found no tangible effect of biotic supplements on urea as compared with control (SMD −0.02; 95% CI −0.25, 0.20; Figure 5). This analysis had a mild level of heterogeneity (*Chi*² = 11.88, *p* = 0.16; *I*² = 33%) that was nullified after excluding four RCTs [6,15,16,25] performed on HD patients and no apparent publication bias (Supplementary Figure S1).

In two single studies enrolling moderate-to-severe CKD patients [13,20], synbiotic administration significantly reduced serum/plasma p-cresol concentration which, conversely, remained stable in the placebo arm. Similar findings were reported in another study of kidney transplant recipients [23] but were not confirmed by three other RCTs enrolling HD [15,25] as well as stage 4–5 CKD (not on dialysis) patients [19].

In one study of 40 HD patients [15], prebiotic treatment reduced plasma levels of free indoxyl sulfate, as compared to control group; conversely, in another study of 33 HD individuals [25], a significant increase in indoxyl sulfate was reported after 12 weeks of probiotic supplementation, with respect to placebo. Finally, no significant variations in this uremic toxin were reported by two more studies performed on moderate-to-severe CKD patients [19,20].

In one study of HD patients [14] a decreasing, although not statistically significant, trend was observed in total indoxyl glucuronide concentrations (−0.11 mg%, *p* = 0.058) after probiotic administration vs. placebo.

One study [19] reported no significant effects of prebiotics on the levels of p-cresyl glucuronide and phenyl-acetyl-glutamine (PAG) but a small, albeit significant reduction of serum trimethylamine N-oxide (TMAO) (*p* = 0.04).

Finally, probiotic supplementation failed to reduce indole-3 acetic-acid (IAA) after 12 weeks of treatment in one trial enrolling ESKD patients on chronic HD [25].

#### 3.6.4. Quality of Life (QoL)

In one RCT [12], the majority of subjects receiving probiotics experienced a considerable improvement in overall QoL (86%, p < 0.05), with respect to those allocated to placebo. 

Conversely, no difference in patient-reported health (KDQOL-36) between the active and control group was observed by three other RCTs [14,15,20].

#### 3.6.5. Nutritional Status

One study [16] found no difference in nutritional status after treatment with synbiotic supplements or placebo (19/20 vs. 11/15 well-nourished individuals; *p* > 0.05). In another study [6], however, probiotic supplementation produced a significant reduction in SGA score (−0.7 ± 2.2 vs. +0.7 ± 1.8; *p* = 0.01) as compared with placebo.

## 4. Discussion

To the best of our knowledge, this systematic review and meta-analysis is the largest and most updated evaluation of the effects of biotic supplements in individuals affected by chronic kidney disease of various severities. Unfortunately, despite information from an adequate number of RCTs and participants (17 studies, 701 individuals), the question as to whether biotics should be advocated in this population setting for improving outcomes remains generally unanswered due to several limitations in the available evidence.

CKD triggers a substantial derangement in the gut microbiota, both in terms of quantity and quality of colonizing bacteria species. This dysbiotic condition is partly consequent to some iatrogenic habits, such as frequent use of antibiotics [26], decreased consumption of dietary fiber [27] or chronic oral iron intake [28]. However, uremia may also alter the normal balance between symbiotic and pathogen bacteria per se [29]. In fact, excess urea due to reduced renal function is secreted into the gastrointestinal tract, where bacterial ureases generate large amounts of ammonia that eventually hamper the growth of commensal microbe species [30]. In renal patients, intestinal dysbiosis also enhances protein fermentation leading to an increased generation of other gut-derived uremic toxins. Similar to urea, such toxins accumulate in the circulation due to a reduced clearance capacity by the failing kidney or, in chronic ESKD dialysis patients, due to the scarce capacity of hemodialysis biomembranes to remove them efficiently. Not surprisingly, serum concentrations of indoxyl sulfate and p-cresyl sulfate have been found to be inversely correlated with residual renal function in CKD individuals [31] and higher levels of indoxyl sulfate and p-cresol have been observed among chronic HD patients with colons, as compared to those without [32]. Although several animal and small uncontrolled clinical studies indicate that biotics might be effective in reducing amines, indoles and cresoles by restoring an optimal gut bacterial milieu [23,33,34,35,36], the vast majority of RCTs included in the present review failed to demonstrate efficacy of these supplements to decrease various uremic toxins. However, information in these studies was mostly available in a format that was not suitable to be pooled in cumulative analyses, hence limiting the possibility to draw overall conclusions in a definite manner.

Uremic toxins are also recognized to accelerate renal disease progression and to increase cardiovascular risk. In uremic rats, administration of indoxyl sulfate induces glomerulosclerosis, renal fibrosis and promotes a significant decline in renal function [37]. In various clinical studies, indoxyl sulfate and p-cresyl sulfate were independent predictors of CKD [38,39,40] and were associated with adverse cardiovascular outcomes, particularly in hemodialysis populations [41,42,43].

Another main aim of our review was to ascertain whether modulation of gut microbiota by biotic supplements could improve renal and cardiovascular outcomes of CKD individuals, as suggested by preliminary available evidence [44]. Unfortunately, we found only a single trial reporting on CV morbidity (cerebrovascular accidents) [5] with just one event recorded, while no study provided information on cardiac events or mortality.

Pooled analyses focusing on end-of-treatment eGFR/creatinine clearance or serum creatinine did not show evidence of efficacy of biotics in improving renal function as compared with the control. 

However, despite such analyses, summarized data from a reasonable number of studies had null heterogeneity and no evidence of publication bias; the generalizability of these (negative) findings remains questionable due to the short to very short treatment period and the small, underpowered sample size of the study populations. Finally, no study provided information on other, more standardized indicators of CKD progression, such as doubling of serum creatinine events, need for dialysis or renal transplantation or eGFR slope over time.

Sustained inflammation is a major hallmark of chronic kidney disease, becoming more prominent as renal failure progresses to end-stage kidney disease, requiring chronic dialysis.

Such an inflammatory state is associated with adverse outcomes, including anemia and erythropoietin hyporesponsiveness, malnutrition, impaired quality of life and, above all, exceedingly high cardiovascular disease with increased mortality and hospitalization.

It is now acknowledged that dysbiosis in the intestinal microflora may contribute to systemic inflammation in CKD. This is mostly due to an increased translocation of endotoxins and other uremic toxins into the circulation, with consequent hyper-activation of monocytes, neutrophils and granulocytes leading to an altered balance between pro- and anti-inflammatory cytokines [45]. In patients undergoing chronic peritoneal- or hemo-dialysis, positive correlations have been found between lipopolysaccharides from gut bacteria and C-reactive protein levels [46,47] and subclinical endotoxemia has recently been acknowledged as a relevant cause for inflammation in patients with CKD [48,49]. Biotic administration may also improve the pro-inflammatory status of CKD patients [50]. Our systematic literature search retrieved a considerable number of RCTs (*n* = 13) testing the effects of biotic supplements on inflammatory markers in renal patients. Yet, there was poor agreement among single study findings and the myriad different pro- or anti-inflammatory cytokines analyzed from various studies hampered the possibility of drawing definite conclusions on a cumulative basis. Conversely, a pooled meta-analysis of the only three RCTs with suitable data on C-reactive protein showed no apparent impact of biotics over control on this inflammatory index; such a negative finding, however, could hardly be generalized in a reliable manner given the paucity of evidence available.

Our review has points of strength and limitations that deserve mentioning. Strengths include a comprehensive systematic approach to the existing literature, study selection, data extraction and quality assessment that have all been conducted according to current methodological standards. Furthermore, we intentionally focused only on randomized controlled trials as a way to minimize selection bias and potential confounding factors, and we considered an ample list of outcomes without restrictions on study duration and type of intervention in order to maximize information gathering.

Limitations mostly rely on the small sample size, the heterogeneity in terms of CKD stage, type and duration of treatment and, as briefly alluded to before, the short follow-up of studies included. Study duration was in most cases of few weeks/months and, hence, not adequate to capture statistically significant differences in parameters of renal function or in the occurrence of cardiovascular events. As a result, most studies actually focused on surrogate, rather than hard endpoints and data on some other clinically relevant outcomes, such as quality of life or nutritional status, were sparse or lacking. Heterogeneity in time and methods of measurement of some end-points may represent another key issue. For instance, serum creatinine and urea levels in chronic dialysis patients are notoriously influenced by the type and efficacy of the dialysis technique itself and by the time point (e.g., pre- or post-dialysis session or during a long or short interval between two sessions) in which they were measured. As most studies did not provide this information in full, the generalizability of findings to the whole dialysis population remains problematic.

Finally, the relatively low number of studies finally included in pooled analyses prevented the possibility to perform more complex investigations, such as additional subgroup or meta-regression analyses, in order to identify all potential treatment–effect modifiers or sub-categories of patients that could possibly take benefit from these supplements as compared to others.

In conclusion, data from currently available RCTs do not provide a clear rationale for suggesting a widespread use of biotic supplements for improving outcomes in renal patients. Future, well-designed and adequately powered trials focusing on hard rather than surrogate outcomes (e.g., mortality/morbidity; CKD progression) are hence advocated for clarifying this issue.

## Figures and Tables

**Table 1:** Summary of main characteristics and findings of the RCTs reviewed.

Study, Year (ref.)	Inclusion Criteria Exclusion Criteria	Population Characteristics	Intervention	Control	Duration	Outcome(s)	Results	Notes
Bliss et al., 1996 [10]	CKD patients underwent low-protein diet for ≥4 months Liver diseases, HD, renal transplantation, active gastrointestinal bleeding, pregnancy or lactating	*n* = 16 Men (%) = 63 Urea (mg/dL) = 50 ± 6 SCr (mg/dL) = 4.4 ± 0.8	Prebiotics (Gum arabic fiber, 25g twice/daily) (*n* = 16)	Placebo (*n* = 16)	Eight weeks	SCr (mg/dL)	End of treatment, 4.5 ± 3.2 vs. 4.7 ± 3.6 in prebiotic vs. placebo group (*p* = 0.12)	Single blind, cross-over Four drop-outs; per-protocol analysis performed
						Urea (mg/dL)	End of treatment, 44 ± 20 vs. 52 ± 32 in prebiotic vs. placebo group (*p* < 0.05)	
Younes et al., 2006 [11]	CKD patients underwent a restrictive protein diet (0.8g/kg/day)	*n* = 9 Age (year) = 67.7 ± 11.5 Men (%) = 33 Urea (mmol/L) = 25 ± 5 CrCl (mL/min) = 25 ± 5	Prebiotics (Fermentable carbohydrate 40 g/day) (*n* = 9)	Standard treatment (*n* = 9)	10 weeks	CrCl (mL/min)	End of treatment, 24.2 ± 13.9 vs. 22.6 ± 12.2 in prebiotic vs. control group (*p* > 0.05)	Open label, cross-over Prebiotic supplementation consisted of 25 g of whole-meal bread, 4.5 g inulin and 10.5 g crude potato starch
						SCr (µmol/L)	End of treatment, 339 ± 146 vs. 357 ± 143 in prebiotic vs. control group (*p* > 0.05)	
						Urea (mmol/L)	End of treatment, 20.2 ± 8.2 vs. 26.1 ± 8.7 in prebiotic vs. control group (*p* < 0.05)	
Ranganathan et al., 2010* [12] Ranganathan et al., 2009	Stage 3–4 CKD patients Antibiotic treatment within 14 days before screening, drugs or alcohol dependence, HIV, liver disease, any medical, psychiatric, debilitating disease, anticoagulant therapy, pregnancy	*n* = 46 Age (year) = ~56 Men (%) = 67 DM (%) = 41	Probiotics (two capsules thrice/daily) (*n* = 46)	Placebo (*n* = 46)	Six months	SCr (μmol/L)	End of treatment, 388.52±229.85 vs. 414.04±342.34 in probiotic vs. placebo group (*p* = 0.23)	Double blind, cross-over Each capsule (15 billion CFU) of probiotic supplementation contained *L. acidophilus*, *B. longum* and *S. thermophilus* Sixteen drop-outs; per-protocol analysis performed
						BUN (µmol/L)	End of treatment, 23.82 ± 12.01 vs. 25.89 ± 15.14 in probiotic vs. placebo group (*p* = 0.039)	
						C-reactive protein (mg/L) (*n* = 13 pts)	End of treatment, mean change −5.32 ± 19.7 vs. 8.55 ± 20.1 in probiotic vs. placebo group (*p* = 0.24)	
						QoL	Improvement of QoL (1.54 ± 1.25) during probiotic group (*p* < 0.001)	
Guida et al., 2014 [13]	Stage 3–4 CKD patients Renal transplant, severe infections and malnutrition, DM, malignancy, food intolerance, autoimmune disorders	*n* = 30 Age (year) = 59.5 ± 13.1 Men (%) = 87 BMI (Kg/m^2^) = ~27.5 eGFR (mL/min) = ~29.2	Synbiotics (5g powder packets thrice/daily) (*n* = 18)	Placebo (*n* = 12)	Four weeks	eGFR (mL/min)	No difference between groups	Double blind Synbiotic formulation consisted of probiotic supplement (*L. plantarum*, 5 billion CFU, *L. casei subsp. Rhamnosus*, 2 billion CFU, *L. gasseri*, 2 billion CFU, *Bifidobacterium infantis*, 1 billion CFU, *Bifidobacterium longum*, 1 billion CFU, *L. acidophilus*, 1 billion CFU, *L. salivarius*, 1 billion CFU, *L. sporogenes*, 1 billion CFU and *Streptococcus thermophilus*, 5 billion CFU) and prebiotic inulin (2.2 g) and tapioca-resistant starch (1.3 g)
						Total p-cresol (µg/mL)	End of treatment, 0.8 (IQR 0.3–3.7) vs. 3.9 (IQR 3.2–5.8) in synbiotic vs. placebo group (*p* < 0.05)	
Ranganathan et al., 2014 [14]	HD patients HIV, liver disease, drugs or alcohol dependence, anticoagulant therapy, medical debilitating disorder, pregnancy	*n* = 22 Age (year) = 54 (29–79) Men (%) = 27 SBP (mmHg) = 148 DBP (mmHg) = 76	Probiotics (two capsules /thrice daily) (*n* = 22)	Placebo (*n* = 22)	Six months	C-reactive protein (mg/L)	No difference between groups	Double blind, crossover Each capsule of probiotic formulation contained 30 billion CFU of *S. thermophilus*, *L. acidophilus* and *B. longum* Six drop-outs; per-protocol analysis performed
						Total indoxyl glucuronide (mg%)	No difference between groups	
						QoL-36 score	No difference between groups	
Sirich et al., 2014 [15]	HD patients Urea clearance >2 mL/min, active gastrointestinal disease, use of antibiotics within four weeks before study entry	*n* = 40 Age (year) = ~56 Men (%) = ~60 HD vintage (year) = ~4 BMI (Kg/m^2^) = ~29 DM (%) = ~45	Prebiotics (resistant starch, up to two sachets/day) (*n* = 20)	Placebo (*n* = 20)	Six weeks	Urea (mg/dL)	End of treatment, 56 ± 14 vs. 60 ± 19 in prebiotic vs. placebo group (*p* > 0.05)	Single blind Fiber sachets contained 15 g of high-amylose corn starch (40% digestible and 60% resistant starch); placebo sachets contained 15 g of waxy corn starch Two patients in control group dropped out because of side effects; no side effects in treatment group Sixteen drop-outs (eight in each group; per-protocol analysis performed)
						C-reactive protein (mg/dL)	End of treatment, 1.1 ± 1.6 vs. 0.8 ± 1.1 in prebiotic vs. placebo group (*p* > 0.05)	
						Free indoxyl sulfate (mg/dL)	End of treatment, 0.25 ± 0.17 vs. 0.28 ± 0.15 in prebiotic vs. placebo group (*p* > 0.05)	
						Total indoxyl sulfate (mg/dL)	End of treatment, 2.9 ± 1.4 vs. 3.1 ± 1.2 in prebiotic vs. placebo group (*p* > 0.05)	
						Free p-cresol (mg/dL)	End of treatment, 0.21 ± 0.14 vs. 0.23 ± 0.14 in prebiotic vs. placebo group (*p* > 0.05)	
						Total p-cresol (mg/dL)	End of treatment, 2.9 ± 1.6 vs. 3.1 ± 1.4 in prebiotic vs. placebo group (*p* > 0.05)	
						QoL-36 score	No difference between groups	
Firouzi et al., 2015 [7]	Type 2 diabetic, mild CKD patients Use of insulin, antibiotics and/or other medication, acute or chronic disease other than DM, hyperlipidemia and hypertension	*n* = 136 Age (year) = 53.5 ± 8.5 Men (%) = 52 BMI (kg/m^2^) = ~29 eGFR (mL/min) = ~74 Urea (mmol/L) = ~4.1 SCr (µmol/L) = ~71	Probiotics (60 billion CFU/day) (*n* = 68)	Placebo (*n* = 68)	12 weeks	eGFR (mL/min)	End of treatment, 73.07 ± 17.13 vs. 68.89 ± 13.55 in probiotic vs. placebo group (*p* = 0.15)	Double-blind Probiotic supplementation consisted of *L. acidophilus*, *L. casei*, *L. lactis*, *Bifidobacterium bifidum, longum* and *infantis* Higher incidence of side effects in probiotic (8.7%) than control group (3.7%) Thirty-five drop outs; (15 in placebo and 20 in probiotic group); ITT and per-protocol analyses performed
						SCr (µmol/L)	End of treatment, 72.26 ± 19.73 vs. 75.17 ± 18.93 in probiotic vs. placebo group (*p* = 0.3)	
						Urea (mmol/L)	End of treatment, 4.04 ± 1.04 vs. 4.24 ± 1.14 in probiotic vs. placebo group (*p* < 0.05)	
Viramontes-Horner et al., 2015 [16]	HD patients, three times/week, for at least three months Current use of probiotics for other reasons, omega-3 fatty acids, pentoxifylline, immunosuppressive and nonsteroidal anti-inflammatory drugs, cancer, HF, chronic liver diseases, intestinal malabsorption and active infection, previous renal transplant	*n* = 35 Age (year) = ~40 Men (%) = ~76.5 HD vintage (year) = ~5 BMI (Kg/m^2^) = ~23.5 DM (%) = ~70 HTN (%) = ~50 SCr (mg/dL) = ~10.5	Synbiotics (11 million CFU/day + 2.31g inulin) (*n* = 20)	Placebo (*n* = 15)	Eight weeks	SCr (mg/dL)	End of treatment, 11.4 (IQR 9.9–13.0) vs. 10.4 (IQR 9.0–13.2) in synbiotic vs. placebo group (*p* > 0.05)	Double blind Synbiotic formulation consisted of probiotic supplement (*L. acidophilus* and *Bifidobacterium lactis*) and prebiotic inulin (2.31 g), omega-3 fatty acids and complex B-vitamins (1.5 g) Patients underwent nutritional counselling consisting of energy (30–35 kcal/kg/day) and protein (1.1–1.2 g/kg/day) intakes and potassium, phosphorus, sodium restriction Seven drop-outs; per-protocol analysis performed
						Urea (mg/dL)	End of treatment, 148.6 ± 41.6 vs. 131.5 ± 43.8 in synbiotic vs. placebo group (*p* > 0.05)	
						C-reactive protein (mg/dL)	End of treatment, 6.3 (IQR 1.8–11.3) vs. 5.0 (IQR 0.6–9.9) in synbiotic vs. placebo group (*p* > 0.05)	
						TNF-α (pg/mL)	End of treatment, 2.9 (IQR 0.9–6.7) vs. 3.1 (IQR 0.0–3.7) in synbiotic vs. placebo group (*p* > 0.05)	
						IL-6 (pg/mL)	End of treatment, 2.0 (IQR 1.2–3.9) vs. 0.6 (IQR 0.2–3.6) in synbiotic vs. placebo group (*p* > 0.05)	
						Nutritional status (SGA)	End of treatment, 1/20 vs. 4/15 had mild to moderate malnutrition in synbiotic vs. placebo group (*p* > 0.05) End of treatment, 19/20 vs. 11/15 were well nourished in synbiotic vs. placebo group (*p* > 0.05)	
Wang et al., 2015 [17]	PD patients with eGFR <15 mL/min/1.73 m^2^ Infectious diseases in the previous month, autoimmune diseases, use of immunosuppressive agents or antibiotics within one month prior to enrollment, pregnancy	*n* = 39 Age (year) = ~52 Men (%) = ~46 PD vintage (mo) = ~42 BMI (Kg/m^2^) = ~23 DM (%) = ~21 HTN (%) = ~81.3 CAD (%) = ~21 SCr (mg/dL) = ~12.5 Urea (mg/dL) = ~58	Probiotics (4 billion CFU/day) (*n* = 21)	Placebo (*n* = 18)	Six months	CrCl (mL/min/1.73 m^2^)	End of treatment, 1.59 (IQR 0.85–2.93) vs. 1.24 (IQR 0.50–2.74) in probiotic vs. placebo group (*p* > 0.05)	Double blind One capsule of probiotics consisted of 1 billion CFU per bacterium strain (*Bifidobacterium bifidum, catenulatum*, *longum* and *L. plantarum*) Eight drop-outs; per protocol analysis performed
						SCr (mg/dL)	End of treatment, 11.76 (IQR 9.55–13.86) vs. 12.84 (IQR 11.84–14.23) in probiotic vs. placebo group (*p* > 0.05)	
						Urea (mg/dL)	End of treatment, 57.0 (IQR 50.0–63.0) vs. 55.5 (IQR 48.0–71.0) in probiotic vs. placebo group (*p* > 0.05)	
						TNF-α (pg/mL)	End of treatment, 0.74 (IQR 0.41–1.29) vs. 0.74 (IQR 0.18–2.22) in probiotic vs. placebo group (*p* > 0.05)	
						IFN-γ (pg/mL)	End of treatment, 7 (IQR 4–12) vs. 8.67 (IQR 2–18.66) in probiotic vs. placebo group (*p* > 0.05)	
						IL-5 (pg/mL)	End of treatment, 9.19 (IQR 7.68–12.61) vs. 9.6 (IQR 7.99–12.6) in probiotic vs. placebo group (*p* > 0.05)	
						IL-6 (pg/mL)	End of treatment, 1.12 (IQR 0.75–3.93) vs. 0.95 (IQR 0.11–1.7) in probiotic vs. placebo group (*p* > 0.05)	
						IL-10 (pg/mL)	End of treatment, 15.97 (IQR 13.47–23.17) vs. 12.69 (IQR 10.25–20.02) in probiotic vs. placebo group (*p* > 0.05)	
						IL-17 (pg/mL)	End of treatment, 1.61 (IQR 0.98–2.2) vs. 2.13 (IQR 1.61–3.8) in probiotic vs. placebo group (*p* > 0.05)	
Dehghani et al., 2016 [5]	Stage 3–4 CKD patients HD, use of antibiotics and lactulose two weeks before study entry, alcohol dependence, hepatitis or HIV infection, pregnancy	*n* = 66 Age (yr)= 61.0 ± 7.65 Men (%) = 75.8 BMI (Kg/m^2^) = 28.5 ± 4.1 DM (%) = 98.5 HTN (%) = 84.6 SCr (mg/dL) = ~2.1 eGFR (mL/min/1.73 m^2^) = ~41.4 Urea (mg/dL) = ~39	Synbiotics (two capsules/twice daily) (*n* = 31)	Placebo (*n* = 35)	Six weeks	Non-fatal CV events	1/31 vs. 0/35 in synbiotic vs. placebo group (*p* > 0.05)	Double blind Two capsules (500 mg) of synbiotic supplement consisted of seven strains of probiotics (*L. casei*, *L. acidophilus*, *L. bulgarigus*, *L. rhamnosus*, *Bifidobacterium breve*, *longum*, *Streptococcus thermophilus*) and prebiotic fructo-oligosaccharides Nine drop-outs; per protocol analysis performed
						eGFR (mL/min/1.73 m^2^)	End of treatment, 43.25 ± 17.49 vs. 39.51 ± 17.64 in synbiotic vs. placebo group (*p* = 0.90)	
						SCr (mg/dL)	End of treatment, 1.90 ± 0.70 vs. 2.18 ±1 .14 in synbiotic vs. placebo group (*p* = 0.15)	
						Urea (mg/dL)	End of treatment, 36.14 ± 20.52 vs. 39.62 ± 27.56 in synbiotic vs. placebo group; *p* = 0.006	
Pavan et al., 2016 [18]	Stage 3–5 CKD patients not on dialysis	*n* = 24 Age (year) = 57.8 ± 7.11 Men (%) = 66.6 Weight (Kg) = 59.2 ± 8.1 BMI (Kg/m^2^) = 22±3.2 DM (%) = 62.5 HTN (%) = 16.7 SCr (mg/dL) = 4.4±0.7	Synbiotics (three tablets/day) (*n* = 12)	Standard therapy (*n* = 12)	Six months	eGFR (mL/min/1.73 m^2^)	GFR declined more rapidly in control than in synbiotic group (−11.6 ± 8.6 vs. −3.4±4.6 per year) (*p* < 0.001)	Open label One tablet of synbiotic supplementation consisted of 15 billion CFU of each bacterium strain (*Streptococcus thermophilus*, *L. acidophilus* and *Bifidobacteria longum*) and 100 mg of prebiotic fructo-oligosaccharides Patients underwent low protein diet (<0.6 g/kg/day)
						SCr (mg/dL)	End of treatment, 4.45 ± 0.30 vs. 4.3 ± 0.31 in synbiotic vs. placebo group (*p* > 0.05)	
Poesen et al., 2016 [19]	CKD patients (eGFR 15–45 mL/min/1.73 m^2^) not on dialysis Gastro-intestinal disease, inflammatory bowel disease, malignancy, previous colorectal surgery and insulin dependent DM, use of antibiotics, prebiotics or probiotics in the previous four weeks before study entry	*n* = 40 Age (year) = 70 ± 6 Men (%) = 70 BMI (Kg/m^2^) = 28.7 ± 5 SCr (mg/dL) = 1.98 (1.60–2.18) eGFR (mL/min/1.73 m^2^) = 33 (27–38) Proteinuria (g/d) = 0.161 (0.078–0.498) Urea (mg/dL) = 65.5 (51.0–75.5)	Prebiotics (Arabinoxylan oligo-saccharides 10g/twice daily) (*n* = 23)	Placebo (*n* = 17)	Four weeks	Urea (mg/dL)	No difference between groups	Double blind, crossover One drop-out (nausea during prebiotic treatment); ITT analysis performed
						p-cresol (μmol/L)	No difference between groups	
						p-cresyl glucuronide (μmol/L)	No difference between groups	
						indoxyl sulfate (μmol/L)	No difference between groups	
						trimethylamine N-oxide (μmol/L)	Treatment effect (prebiotic vs. placebo) −0.237; 95% CI −0.464, −0.010; *p* = 0.04)	
						phenyl-acetyl-glutamine (μmol/L)	No difference between groups	
SYNERGY 2016* [20,21,22]	Moderate to severe (pre-HD), hypertensive CKD patients Previous renal transplant, bowel resection or bowel radiation recipient, bowel syndrome, Crohn disease or ulcerative colitis, likely to receive a transplant or progress to dialysis within six months, pre, probiotics or antibiotic use within one month, or change in immunosuppressant dose within six months before study entry	*n* = 31 Age (year) = 69 ± 9 Men (%) = 61 BMI (Kg/m^2^) = 28 ± 6 eGFR (mL/min/1.73 m^2^) = 25 ± 8 Proteinuria (mg/day) = 296 (168–1100) Albuminuria (mg/day) = 97 (21–677)	Synbiotic supplements (15 g powder + two capsules/day) (*n* = 13)	Placebo (*n* = 18)	16 weeks	eGFR (mL/min/1.73 m^2^)	End of treatment, 24 ± 8 vs. 24 ± 8 in synbiotic vs. placebo group (*p* = 0.67)	Double blind, crossover Participants underwent a two week run-in period (dietary education with stable protein and fiber intakes) before randomization Synbiotic formulation consisted of 15 g prebiotic (a combination of high–molecular weight inulin, fructo-oligosaccharides and galacto-oligosaccharides) and 90 billion CFU probiotic component (capsule) including nine different strains across the *Lactobacillus*, *Bifidobacteria* and *Streptococcus* genera Six drop-outs; per protocol analysis performed
						SCr (µmol/L)	End of treatment, 231 ± 75 vs. 233 ± 74 in synbiotic vs. placebo group (*p* = 0.94)	
						Proteinuria (mg/day)	End of treatment, 369 (IQR 162–1550) vs. 323 (IQR 169–1150) in synbiotic vs. placebo group (*p* = 0.20)	
						Albuminuria (mg/day)	End of treatment, 112 (IQR 16–758) vs. 111 (IQR 12–594) in synbiotic vs. placebo group (*p* > 0.05)	
						IL-1β (pg/mL)	End of treatment, 0.8 ± 0.7 vs. 0.8 ± 0.6 in synbiotic vs. placebo group (*p* = 0.98)	
						IL-6 (pg/mL)	End of treatment, 2.2 ± 0.9 vs. 2.0 ± 0.8 in synbiotic vs. placebo group (*p* = 0.40)	
						IL-10 (pg/mL)	End of treatment, 3.6 ± 2.0 vs. 3.6 ± 2.1 in synbiotic vs. placebo group (*p* = 0.84)	
						TNF-α (pg/mL)	End of treatment, 2.2 ± 0.8 vs. 2.0 ± 0.7 in synbiotic vs. placebo group (*p* = 0.09)	
						Free indoxyl sulfate (µmol/L)	End of treatment, 0.6 (IQR 0.4–0.8) vs. 0.5 (IQR 0.4–1.0) in synbiotic vs. placebo group (*p* = 0.20)	
						Total indoxyl sulfate (µmol/L)	End of treatment, 15 (IQR 10–26) vs. 16 (IQR 12–27) in synbiotic vs. placebo group (*p* = 0.12)	
						Free p-cresol (µmol/L)	End of treatment, 2.2 (IQR 0.7–2.8) vs. 2.4 (IQR 1.1–3.4) in synbiotic vs. placebo group (*p* = 0.34)	
						Total p-cresol (µmol/L)	End of treatment, 75 (IQR 36–101) vs. 93 (IQR 54–136) in synbiotic vs. placebo group (*p* = 0.03)	
						Physical patient-reported health score	End of treatment, 35 ± 11 vs. 37 ± 10 in synbiotic vs. placebo group (*p* = 0.23)	
						Mental patient-reported health score	End of treatment, 51 ± 10 vs. 52 ± 9 in synbiotic vs. placebo group (*p* = 0.75)	
Guida et al., 2017 [23]	Kidney transplanted patients with stable graft function Acute rejection or infection in the previous three months, DM, malignancy, pregnancy, food intolerance, autoimmune disorders, severe malnutrition or clinical conditions requiring artificial feeding	*n* = 34 Age (year) = ~50.6 Men (%) = ~82.3 Weight (Kg) = ~75.6 WC (cm) = ~94.7 BMI (Kg/m^2^) = ~25.4 eGFR (mL/min/1.73 m^2^) = ~54.5	Synbiotic supplements (three times/day) (*n* = 22)	Placebo (*n* = 12)	Four weeks	eGFR (mL/min/1.73 m^2^)	End of treatment, 53.5 ± 16.0 vs. 57.3 ± 22.1 in synbiotic vs. placebo group (*p* > 0.05)	Double blind Synbiotic (Probinul Neutro) consisted of probiotic supplement (*L. plantarum*, 5 billion CFU, *L. casei subsp*. *Rhamnosus*, 2 billion CFU, *L. gasseri*, 2 billion CFU, *Bifidobacterium infantis*, 1 billion CFU, *B. longum*, 1 *Streptococcus thermophilus*, 5 billion CFU) and prebiotic inulin (2.2 g) and tapioca-resistant starch (1.3 g) Two drop-outs; per protocol analysis performed
						Total p-cresol (µg/mL)	End of treatment, 2.3 (IQR 0.9–2.72) vs. 4.4 (IQR 3.0–6.4) in synbiotic vs. placebo group; *p* < 0.01	
Miraghajani et al., 2017 [24]	Type 2 diabetic patients with early CKD (proteinuria >300 mg/day and eGFR >90 mL/min) Intolerance to soy milk, smoking, alcoholism, recent antibiotic therapy and use of supplements containing vitamins and minerals, inflammatory bowel disease, infection, liver disease and rheumatoid arthritis	*n* = 40 Age (year) = ~55.2 Men (%) = ~47.5 Weight (Kg) = ~71 BMI (Kg/m^2^) = ~26.5	Probiotics (2 billion CFU/day) (*n* = 20)	Placebo (*n* = 20)	Eight weeks	Progranulin (ng/mL)	End of treatment, 180.90 ± 69.25 vs. 399.56 ± 105.20 in probiotic vs. control group; *p* = 0.01	Double blind Participants received individualized dietary counselling (restricted dietary protein, sodium, and potassium intake) before randomization to a diet containing 200 mL/day probiotic soy milk (fortified with 20 million CFU/mL of *L. plantarum*) or conventional soy milk
Soleimani et al., 2017 [6]	Diabetic HD patients, three times/week, for at least one year Intestinal diseases, use of probiotic supplements, prebiotic, antioxidant and anti-inflammatory supplements (vitamin E, C and omega-3 fatty acids), antibiotics and immunosuppressive medications within three months before enrolment, pregnancy	*n* = 60 Age (year) = ~56.7 Men (%) = 66.7 Weight (Kg) = ~68.3 BMI (Kg/m^2^) = ~26.3 HD vintage (year)= ~3.5 HTN (%) = 96.7 SCr (mg/dL) = ~7.6 eGFR (mL/min/1.73 m^2^) = ~2.35 hs-CRP (ng/mL) = ~7672 Urea (mg/dL) = ~59.2 CVD (%) = ~21.6 CAD (%) = ~78.3	Probiotics (6 billion CFU/day) (*n* = 30)	Placebo (*n* = 30)	12 weeks	eGFR (mL/min/1.73 m^2^)	End of treatment, 2.54 ± 1.16 vs. 2.25 ± 0.93 in probiotic vs. placebo group (*p* = 0.77)	Double blind Probiotic supplementation consisted of *L. acidophilus*, *L. casei* and *Bifidobacterium bifidum*, 2 billion CFU/day per strain Five drop-outs; ITT analysis performed
						SCr (mg/dL)	End of treatment, 7.2 ± 2.6 vs. 7.7 ± 2.9 in probiotic vs. placebo group (*p* = 0.73)	
						Urea (mg/dL)	End of treatment, 63.9 ± 26.0 vs. 52.3 ± 12.7 in probiotic vs. placebo group (*p* = 0.96)	
						hs-C-reactive protein (ng/mL)	End of treatment, 6110 ± 4812.5 vs. 7555.7 ± 5316.2 in probiotic vs. placebo group; *p* = 0.03	
						Subjective global assessment (SGA) score	End of treatment, 8.8 ± 2.0 vs. 10.2 ± 3.7 in probiotic vs. placebo group; *p* = 0.01	
Borges et al., 2018 [25]	HD patients, three times/week, for at least six months Inflammatory and autoimmune diseases, AIDS, cancer, smokers, HD with central catheter access, pregnancy, use of catabolic drugs, antioxidant vitamin supplements, pre-, pro-, synbiotic and antibiotics in the previous three months before study entry	*n* = 33 Age (year) = ~52 Men (%) = ~63.6 WC (cm) = ~92 BMI (Kg/m^2^) = ~25.2 HD vintage (month) = ~48.3	Probiotics (90 billion CFU/day) (*n* = 16)	Placebo (*n* = 17)	12 weeks	SCr (mg/dL)	End of treatment, 9.6 ± 7.7 vs. 10.3 ± 0.6 in probiotic vs. placebo group (*p* = 0.66)	Double blind Probiotic supplementation consisted of *Streptococcus thermophilus, L. acidophilus* and *Bifidobacteria longum* Tirtheen drop-outs; per-protocol analysis performed
						Urea pre-HD (mg/dL)	End of treatment, 172.6 ± 45.0 vs. 155.9 ± 38.6 in probiotic vs. placebo group (*p* = 0.37)	
						Urea post-HD (mg/dL)	End of treatment, 51.3 ± 19.7 vs. 49.5 ± 12.7 in probiotic vs. placebo group (*p* = 0.54)	
						C-reactive protein (mg/dL)	End of treatment, 5.5 (95% CI 2.8, 11.7) vs. 1.7 (95% CI 0.8, 6.4) in probiotic vs. placebo group (*p* = 0.47)	
						IL-6 (pg/mL)	End of treatment, 38.4 ± 20.1 vs. 30.3 ± 18.5 in probiotic vs. placebo group (*p* = 0.91)	
						Total indoxyl sulfate (mg/L)	End of treatment, 36.5 ± 15 vs. 42.5 ± 11.0 in probiotic vs. placebo group (*p* = 0.60)	
						Total p-cresol (mg/L)	End of treatment, 46.3 ± 32.7 vs. 57.5 ± 29.8 in probiotic vs. placebo group (*p* = 0.83)	
						Total indole-3 acetic-acid (µg/L)	End of treatment, 456.8 ± 199 vs. 744.9 ± 309 in probiotic vs. placebo group (*p* = 0.45)	

Legend AIDS: acquired immune deficiency syndrome; BMI: body mass index; BUN: blood urea nitrogen; CAD: coronary artery disease; CFU: colony forming units; CI: confidence interval; CKD: chronic kidney disease; CrCl: creatinine clearance; hs-CRP: high sensitive C-reactive protein; CV: cardiovascular; CVD: cardiovascular disease; DBP: diastolic blood pressure; DM: diabetes mellitus; eGFR: estimated glomerular filtration rate; HD: hemodialysis; HF: heart failure; HIV: human immunodeficiency virus; HTN: hypertension; IFN: interferon; IL: interleukin; IQR: interquartile range; ITT: intention to treat; PD: peritoneal dialysis; QoL: quality of life; RCT: randomized clinical trial; SBP: systolic blood pressure; SCr: serum creatinine; SGA: subjective global assessment; TNF: tumor necrosis factor; WC: waist circumference; *: main study.

**Table 2:** Risk of bias in randomized controlled trials.

Study, Year (ref.)	Random Sequence Generation	Allocation Concealment	Blinding of Participants and Personnel	Blinding of Outcome Assessors	Incomplete Outcome Data	Selective Reporting	Other Sources of Bias
Bliss et al., 1996 [10]	Unclear (not stated)	Low risk (“placebo and prebiotic were similar in appearance, taste and viscosity”)	High risk (single blind)	Unclear (not stated)	Low risk (four drop-outs, 20%; per-protocol analysis performed)	Low risk	None known
Younes et al., 2006 [11]	Unclear (not stated)	Unclear (not stated)	High risk (open label)	Unclear (not stated)	Low risk (no drop-out)	Low risk	None known
Ranganathan et al., 2010 [12]	Unclear (not stated)	Low risk (“placebo and probiotic were similar in color, size and visual look”)	Low risk (double blind)	Unclear (not stated)	High risk (16 drop-outs, 26%; per-protocol analysis performed)	Low risk	High risk of funding bias (“Kibow Biotech has funded publication of the article”)
Guida et al., 2014 [13]	Low risk (computer-generated random binary list)	Low risk (“placebo and synbiotic were comparable in color, texture and taste”)	Low risk (double blind)	Unclear (not stated)	Low risk (no drop-out)	Low risk	Low risk of funding bias (“No external funding for the study”)
Ranganathan et al., 2014 [14]	Unclear (not stated)	Unclear (not stated)	Low risk (double blind)	Unclear (not stated)	High risk (six drop-outs, 21%; per-protocol analysis performed)	High risk (insufficient information on uremic toxins and QoL)	High risk of funding bias (“Kibow Biotech financed the clinical investigation; part of the data was also obtained in Kibow’s own equipped research laboratories”)
Sirich et al., 2014 [15]	Low risk (permuted-block randomization)	Low risk (“fiber supplements and control were provided as white powder in identical sachets”)	High risk (single blind)	Unclear (not stated)	High risk (16 drop-outs, 28.5%; per-protocol analysis performed)	Low risk	High risk of funding bias (“T.L.S. was supported by a Mitsubishi Tanabe Pharma Corporation, National Kidney Foundation Fellowship for the Study of Uremia”)
Firouzi et al., 2015 [7]	Low risk (“computer-generated random-blocks of four and eight in order to allow having exact number of 68 in each group”)	Low risk (“probiotic and placebo sachets were identical in weight, appearance, texture, nutritional value and smell”)	Low risk (double blind)	Unclear (not stated)	High risk (35 drop-outs, 29% vs. 22%; ITT and per-protocol analyses performed)	Low risk	Low risk of funding bias (“Hexbio® B-Crobes Laboratory Sdn. Bhd. did not interfere with the decision to exploit research results”)
Viramontes-Horner et al., 2015 [16]	Unclear (not stated)	Low risk (“placebo and symbiotic supplement had identical color, size and flavor”)	Low risk (double blind)	Unclear (not stated)	Low risk (seven drop-outs, 16%; per-protocol analysis performed)	Low risk	High risk of funding bias (“FMC worked for Nutrimentos Inteligentes, S.A. de C.V., the funders of the study, providing methodological and statistical support”)
Wang et al., 2015 [17]	Low risk (computer-generated random-number table sequence)	Low risk (“allocations contained in opaque, sequentially numbered, sealed envelopes”)	Low risk (double blind)	Unclear (not stated)	Low risk (eight drop-outs, 17%; per-protocol analysis performed)	Low risk	None known
Dehghani et al., 2016 [5]	Unclear (not stated)	Low risk (“placebo and synbiotic produced in similar color and appearance; patients and researcher were not informed of the boxes’ codes)	Low risk (double blind)	Unclear (not stated)	Low risk (nine drop-outs, 12%; per-protocol analysis performed)	Low risk	None known
Pavan et al., 2016 [18]	Unclear (not stated)	Unclear (not stated)	High risk (open label)	Unclear (not stated)	Unclear (not stated)	Low risk	None known
Poesen et al., 2016 [19]	High risk (“randomization performed by the sealed envelope system; the study nurse randomly opened a preformed envelope containing the allocated treatment regimen”)	Low risk (“prebiotic or placebo provided in identical vials and boxes, labeled with a numerical code, unique to treatment allocation”)	Low risk (double blind)	Unclear (not stated)	Low risk (one drop-out; ITT analysis performed)	Low risk	Low risk of funding bias (“Funders had no role in study design, data collection and analysis, decision to publish or preparation of the manuscript”)
SYNERGY 2016 [20]	Low risk (“A computer–generated randomization list with blocks of size 2 produced by an external statistical consultant”)	Low risk (“allocation concealed to researchers and participants; supplements were packed off-site with a generic label, supplement A or B”)	Low risk (double blind)	Unclear (not stated)	Low risk (six drop-outs, 16%; per-protocol analysis performed)	Low risk	High risk of funding bias (“Study funded through a project grant from the Princess Alexandra Private Practice Trust Fund (PPTF). M.R. received the Princess Alexandra PPTF Postgraduate Scholarship”)
Guida et al., 2017 [23]	Low risk (“randomization, 2:1, conducted using a computer-generated random binary list,”)	Low risk (“synbiotic and placebo powders were comparable in color, texture, and taste”)	Low risk (double blind)	Unclear (not stated)	Low risk (two drop-outs, 5.5%; per-protocol analysis performed)	Low risk	None known
Miraghajani et al., 2017 [24]	Low risk (allocation by randomly permuted blocks)	Low risk (“concealed envelopes with consecutive numbers were locked in a drawer and withdrawn in numerical order”)	Low risk (double blind)	Low risk (“outcome’s assessors and analyses’ performers were masked to group assignment”)	Low risk (eight drop-outs, 16.6%; per-protocol analysis performed)	Low risk	High risk of funding bias (“Financial support provided by the Security Research Center, Isfahan University of Medical Sciences, Isfahan, Iran”)
Soleimani et al., 2017 [6]	Low risk (“randomization conducted using computer-generated random numbers”)	Unclear (not stated)	Low risk (double blind)	Unclear (not stated)	Low risk (five drop-outs, 8%; ITT analysis performed)	Low risk	None known
Borges et al., 2018 [25]	High risk (manually generated simple randomization)	Low risk (“participant and researcher were blinded to the contents of bottles containing placebo and probiotic capsules”)	Low risk (double blind)	Low risk (“outcome measurements performed in a blinded manner”)	High risk (13 drop-outs, 28%; per-protocol analysis performed)	Low risk	None known

Legend ITT: intention-to-treat, QoL: quality of life.

## Supplementary Materials

The following are available online at http://www.mdpi.com/2072-6643/10/9/1224/s1.
