# Supplementary material for: Biotic Supplements for Renal Patients: A Systematic Review and Meta-Analysis
Source: Nutrients. 2018 Sep 4;10(9):1224. doi: 10.3390/nu10091224 (PMC6165363; doi:10.3390/nu10091224)
Supplement: Supplementary file 1 [file nutrients-10-01224-s001.zip › Table S1.docx]

**Supplementary Table S1**. Search strategy in CENTRAL, Ovid-Medline and PubMed databases

| **CENTRAL** |
| --- |
| #1 probiotic* or prebiotic* or synbiotic* or lactobacillus or “dried yeast”:ti,ab,kw  #2 MeSH descriptor: [Probiotics] explode all trees  #3 MeSH descriptor: [Prebiotics] explode all trees  #4 MeSH descriptor: [Synbiotics] explode all trees  #5 MeSH descriptor: [Lactobacillus] explode all trees  #6 MeSH descriptor: [Yeast, Dried] explode all trees  #7 #1 or #2 or #3 or #4 or #5 or #6  #8 dialysis:ti,ab,kw  #9 (hemofiltration or haemofiltration):ti,ab,kw  #10 (hemodiafiltration or haemodiafiltration):ti,ab,kw  #11 (end-stage renal or end-stage kidney or endstage renal or endstage kidney):ti,ab,kw  #12 (ESRF or ESKF or ESRD or ESKD):ti,ab,kw  #13 (chronic kidney or chronic renal):ti,ab,kw  #14 (CKF or CKD or CRF or CRD):ti,ab,kw  #15 (CAPD or CCPD or APD):ti,ab,kw  #16 (predialysis or pre-dialysis):ti,ab,kw  #17 MeSH descriptor Kidney Failure, Chronic explode all trees  #18 MeSH descriptor Renal Replacement Therapy explode all trees  #19 MeSH descriptor Renal Insufficiency, Chronic explode all trees  #20 (#8 OR #9 OR #10 OR #11 OR #12 OR #13 OR #14 OR #15 OR #16 OR #17 OR #18 OR #19)  #21 #7 AND #20 |
| **OVID-Medline** |
| 1. exp Renal Dialysis/  2. (hemodialysis or haemodialysis).tw.  3. (hemofiltration or haemofiltration).tw.  4. (hemodiafiltration or haemodiafiltration).tw.  5. dialysis.tw.  6. (CAPD or CCPD or APD).tw.  7. Renal Insufficiency/  8. Kidney Failure/  9. exp Renal Insufficiency, Chronic/  10. Kidney Diseases/  11. Uremia/  12. (end-stage renal or end-stage kidney or endstage renal or endstage kidney).tw.  13. (ESRF or ESKF or ESRD or ESKD).tw.  14. (chronic kidney or chronic renal).tw.  15. (CKF or CKD or CRF or CRD).tw.  16. (predialysis or pre-dialysis).tw.  17. ur?emi$.tw.  18. or/1-17  19. exp probiotic agent  20. exp prebiotic agent  21. exp synbiotic agent  22. exp lactobacillus  23. exp bifidobacterium  24. exp dried yeast  25. (probiotic* or prebiotic* or synbiotic* or lactobacillus or dried yeast or bifidobacteri*).tw.  26. or/19-25  27. 18 and 26 |
| **PubMed** |
| (((("probiotics"[MeSH Terms] OR "probiotics"[All Fields] OR "probiotic"[All Fields]) OR "Fermented"[All Fields]) OR ("lactobacillales"[MeSH Terms] OR "lactobacillales"[All Fields])) OR ("bifidobacterium"[MeSH Terms] OR "bifidobacterium"[All Fields])) OR ("cultured milk products"[MeSH Terms] OR ("cultured"[All Fields] AND "milk"[All Fields] AND "products"[All Fields]) OR "cultured milk products"[All Fields])) OR (("synbiotics"[MeSH Terms] OR "synbiotics"[All Fields] OR "synbiotic"[All Fields]) OR ("prebiotics"[MeSH Terms] OR "prebiotics"[All Fields] OR "prebiotic"[All Fields]))) AND ((chronic kidney disease OR CKD OR chronic renal failure OR chronic renal insufficiency OR CRF OR end stage kidney disease OR ESKD OR end stage renal disease OR ESRD OR dialysis))) |
