# Supplementary figures and images for: Biotic Supplements for Renal Patients: A Systematic Review and Meta-Analysis
Source: Nutrients. 2018 Sep 4;10(9):1224. doi: 10.3390/nu10091224 (PMC6165363; doi:10.3390/nu10091224)

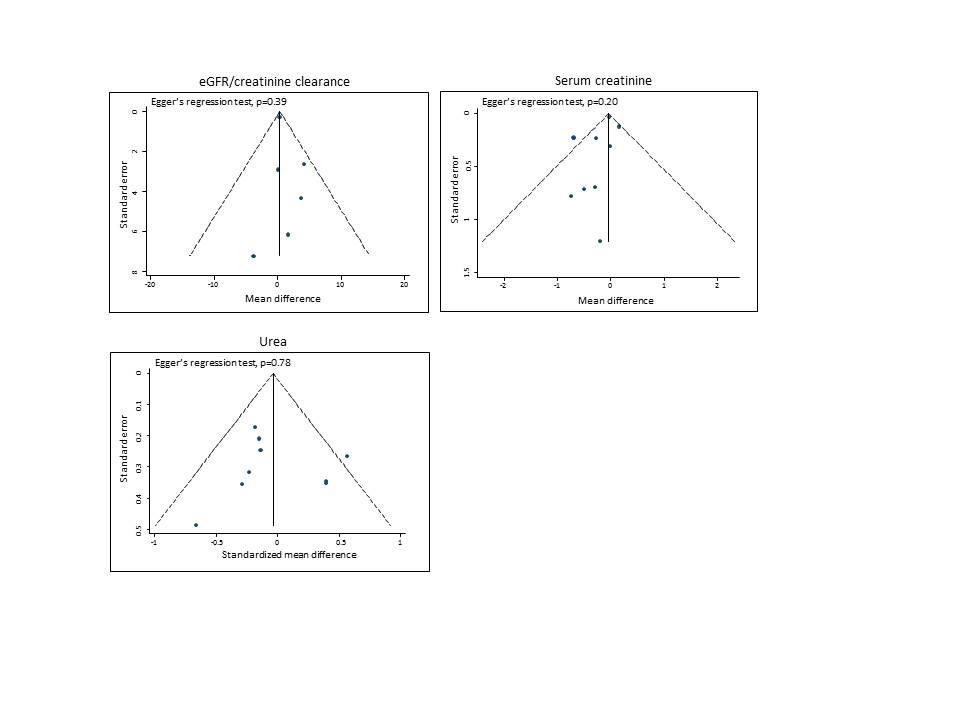

Supplement: Supplementary file 1 [file nutrients-10-01224-s001.zip › Figure S1.tif]
